# Ethical and Legal Implications of Elective Ventilation and Organ Transplantation: “Medicalization” of Dying versus Medical Mission

**DOI:** 10.1155/2014/973758

**Published:** 2014-07-14

**Authors:** Paola Frati, Vittorio Fineschi, Matteo Gulino, Gianluca Montanari Vergallo, Natale Mario Di Luca, Emanuela Turillazzi

**Affiliations:** ^1^Department of Anatomical, Histological, Forensic and Orthopaedic Sciences, University of Rome Sapienza, Viale Regina Elena 336, 00161 Rome, Italy; ^2^Neuromed, Istituto Mediterraneo Neurologico (IRCCS), 86170 Isernia, Italy; ^3^Department of Legal Medicine, University of Foggia, Ospedale Colonnello D'Avanzo, Via degli Aviatori 1, 71100 Foggia, Italy

## Abstract

A critical controversy surrounds the type of allowable interventions to be carried out in patients who are potential organ donors, in an attempt to improve organ perfusion and successful transplantation. The main goal is to transplant an organ in conditions as close as possible to its physiological live state. “Elective ventilation” (EV), that is, the use of ventilation for the sole purpose of retrieving the organs of patients close to death, is an option which offsets the shortage of organ donation. We have analyzed the legal context of the dying process of the organ donor and the feasibility of EV in the Italian context. There is no legal framework regulating the practice of EV, neither is any real information given to the general public. A public debate has yet to be initiated. In the Italian cultural and legislative scenario, we believe that, under some circumstances (i.e., the expressed wishes of the patient, even in the form of advance directives), the use of EV does not violate the principle of beneficence. We believe that the crux of the matter lies in the need to explore the real determination and will of the patient and his/her orientation towards the specific aim of organ donation.

## 1. Introduction

Defining death, like defining life, continues to be a challenge [1]. Death can be considered in terms of medical, legal, ethical, philosophical, societal, cultural, and religious rationales. The medical definition of death is primarily a scientific issue based on the best available evidence [2]. There is a growing consensus that there is a unifying medical concept of death which can be determined by physicians in two ways: (1) by showing the irreversible cessation of all clinical brain functions or (2) by showing the permanent cessation of circulatory and respiratory functions [3].

## 2. The Philosophical Paradox of Explanting a “Live” Organ from a “Dead” Body

The process of the “medicalization of dying” and the practice of transplantation medicine underscore the importance of defining and conceptualizing death and of identifying the moment of death. It is obvious that the issues concerning organ donation and transplantation are closely connected to many other fundamental philosophical, ethical, and legal issues, many of them to do with the definition and criteria of death [4]. What is human death? When does a human being really die? How can we determine that it has occurred?

The issues of defining and determining death have always generated a lively scientific and ethical debate that is brought into sharper focus around the subject of organ donation. For the recovery of organs, is the neurological determination of death (brain death) acceptable in patients whose circulation and respiration are mechanically maintained, or is the cessation of circulation and respiration (circulatory death) necessary? In the first case, is it ethical and/or legal to consider the individual as a potential organ provider and to keep him or her alive by specific vital support devices for the sole purpose of preserving tissue viability and allowing organ explant (once consensus has been obtained), with the subsequent voluntary interruption of vital support? And lastly, is it really necessary to define the legal concept of death in order to proceed to organ transplantation?

There are two additional fundamental ethical premises in meeting the donor's best interests: (i) his/her right to decide with regard to organ donation (which can be delegated to relatives) and (ii) certification of the donor's death. The “dead-donor rule” refers to the widely accepted ethical and juridical norm that governs practices of organ procurement for transplantation: vital organs should be taken only from dead patients. An intense debate has developed on how to verify death, and several ambiguities in both brain and circulatory death determination still need to be resolved. Circulatory-respiratory or brain tests are widely accepted for defining and determining death, but there are still several controversial issues. There are questions that still need a definitive answer, such as whether the whole-brain or brainstem criterion is correct, whether one neurological examination or two should be required, and the minimum duration of asystole which is sufficient for death to be declared in circulatory death [5].

Finally, a critical controversy surrounds the type of allowable interventions to be carried out in patients who are potential organs donors in an attempt to improve organ perfusion and successful transplantation [6]. It concerns the difficult balance between the doctor's commitment to safeguard the patient's health versus unnecessary invasive treatment.

## 3. “Elective Ventilation”: “Medicalization” of Dying versus Medical Mission—The Hierarchy of Ethics

To meet the growing demand for organs, a number of initiatives can be envisaged [7]. To this end, the main goal is to transplant an organ in conditions as close as possible to its physiological live state. Therefore, it is crucial to optimize specific rules establishing when and how an organ can be explanted from a dead or dying patient to be transplanted in a living patient whose life depends on it.

“Elective ventilation” (EV) or “nontherapeutic ventilation” (i.e., the use of ventilation for the sole purpose of retrieving the organs of patients close to death) is an alternative option to offset the shortage of organ donation [8]. This entails targeting patients in a deep irreversible coma for whom death is believed to be imminent and transferring them to intensive care units so that artificial ventilation can be initiated as soon as respiratory arrest occurs, thus preserving the organs until brain death can be established [9]. EV practice is already common in the United States and Spain. However, it was banned by the United Kingdom's Department of Health in 1994 on the grounds that it was unlawful to ventilate a patient for the purpose of harvesting organs, as it did not constitute a procedure undertaken for the patient's benefit, particularly in the absence of patient consent [10].

Thorny ethical issues surround the practice of elective ventilation [11–13], the main one being the potential conflict of (i) the best interests of the “donor” patient (subject to intensive care) versus (ii) the best interests of the patient “recipient” of the transplanted organ (who becomes the final target of the intensive care provided to the “donor” patient).

EV is essentially a question of weighing the patients' best interests. In other words, the medical mission shifts its target, providing intensive care, not for the benefit of the “donor” patient (including the right to die with dignity and not to prolong the death process) but for that of the “recipient” patient.

Therefore, in this case, the doctor chooses according to a two-order hierarchical criterion (i) the best interests of the potential donor patient or (ii) the best interests of the potential recipient patient, for whom the donor patient can be sacrificed. The latter thus becomes the prevailing criterion. Indeed, the interests of the donor lie in his or her right to live or die with dignity (i.e., right to refuse poor quality of life). However, the best interests of the donor, if these entail refusing therapies which are unable to provide a good quality of life or choosing not to prolong the dying process without dignity, are not guaranteed so as to provide the recipient with the best chance to live.

Finally, the practice of EV raises the question of consent [14]. Doubts have been cast on the legality of EV on the grounds that relatives are not permitted to consent to the treatment of an incompetent person when that treatment is not in the patient's best interests. When a patient is transferred to an intensive care unit and subsequent ventilation and circulatory support procedures are initiated solely for the purpose of maintaining the adequate perfusion of organs until retrieval can be arranged, offering no benefit to the patient undergoing these procedures, can the family's consent be considered ethically and legally valid? Can it be assumed that when an individual consciously chooses to become an organ donor, he/she also expects the best care of their organs to ensure successful transplants, thus implicitly agreeing to EV [15]? Can we accept as coherent the argument put forward by Coggon [16] that when a patient wishes to donate, measures, such as EV, which are necessary for organ donation to proceed, serve, rather than deny, the best interests of the patient? Or are these separate matters? Could a patient's advance statement of consent obviate the question and make EV not only ethically acceptable but also lawful [17, 18]?

## 4. The Legal Italian Context of the Dying Process of the Organ Donor

In Italy, the law currently in force (law 578/93 and the Ministerial Decree dated 2008) states the diagnostic criteria for the determination of an individual's death. The premise is a unifying definition of death as the irreversible cessation of all whole-brain functions. Cardiac death is defined when the cardiac arrest lasts long enough to determine the irreversible cessation of all brain functions. Strict diagnostic criteria have to be met until a patient is declared dead.

The Italian system regulating the donation of organs is an “opt-out” one where all citizens over 18 are automatically registered to donate their organs when they die unless they actively decide not to. From an ethical and legal perspective, one must note that an “opt-out” system moves towards the principle of “assumed consent.”

In this Italian regulatory scenario, some ethical and legal issues arise regarding the lawfulness of EV.

First of all, since EV is administered in the interests of a potential recipient and does not fall within the narrow medical interests of the potential donor, Italian physicians have to tackle the issue of the extensive medical procedures that involve significant discomfort and expense to that donor. According to the Italian Code of Medical Ethics “*the physician, also taking into account the patient's will, when this is expressed, must abstain from persisting in diagnostic and therapeutic procedures from which it is not possible to reasonably expect a benefit for the patient's health and/or an improvement in the quality of life*” (art. 16). There is no doubt that the practice of EV does not have the therapeutic care of the patient (suitable donor organ) as its main purpose. Secondly, since potential organ donors who are eligible for receiving EV generally lack the capacity to make decisions as a consequence of their injury, issues arise concerning consent, the legitimacy of the patient's relatives to decide, the decision-making role of the physician, and finally the value of the patient's previously expressed will. The Italian regulatory system does not allow physicians to initiate EV solely on the grounds of the patient's hypothetical consent. An explicit, informed consent is required before any diagnostic/therapeutic act can be performed. Nor does any consent to EV expressed by the patient's relatives have any legal value in the Italian law system unless in the case of a patient under 18 or in the case of an interdict. Family members have no power to consent (or dissent) under Italian law. Therefore, the crux of the question of the feasibility of EV lies in the value of the patient's previously expressed will regarding EV itself. The Italian legal system lacks a specific law on the matter of advance directives and their legal value. Indeed, an official stance expressed by the National Bioethics Committee (NBC) focuses on the medical obligation to pay the utmost attention to the person's will, even if this is expressed in an advance directive. In 2003, the NBC drew up conclusive bioethical recommendations which gave full legitimacy to public advance statements redacted in written form, devoid of any prospect of euthanasia, compiled with the help of a physician, as specific and personalized as possible, and by which the physician should abide even if this is not compulsory [19]. Moreover, the Italian medical code underlines the full significance of an advance directive from a currently incompetent patient, affirming the medical obligation not to elude a previously expressed wish. Many bills have been drafted and presented for approval to the Italian Parliament during past legislatures, in particular regarding the requirements for validity and the possible contents of the patient's previously manifested will and also regarding its potential binding character for the physician who receives it. In the absence of a comprehensive law, what strongly emerge are the principles of deontological codification and of the aforementioned document by the NBC, which postulate the physician's duty to respect the patient's will, even if previously expressed [20]. The value at stake is essentially the right of those who are in full possession of their mental faculties to freely decide. However, several questions remain unsolved, namely, those regarding the limitations and the contents of advance directives. Given that the Italian law (law 91/1999) allows all adult and competent citizens to express their consent/dissent to the donation of organs, might it not be coherent for the same citizens to give their opinion on the possible use of EV to make that donation possible under particular circumstances? For citizens in favor of organ donation, should the agreement to EV be presumed even in the absence of expressed consent? Could an advance directive not allow individuals to specify in advance that under certain circumstances they would consent to elective ventilation merely in order to donate their own organs?

These are the main concerns regarding the practice of EV that will arise in the Italian debate.

In the face of the complex issues mentioned above, it appears natural to raise the question as to whether there is a correct approach to decision-making that can offer assistance to the Italian physicians who are faced daily with the issue of organ donation and elective ventilation.

In the absence of a legal framework regulating the practice of EV, we believe that a reassuring way might be an approach to decision-making which emphasizes patient autonomy.

Patient autonomy should be a central principle and one which physicians ought always to respect. Every action requires that a person has the capacity and opportunity to freely and voluntarily make medical choices, and there is no doubt that even the choice of EV is a medical one. Italian physicians and those responsible for health policy have to recognize and even promote the autonomous actions of the patient in this matter. An increasing number of people, also in Italy, are preparing some form of advance directive to guide their caregivers as to their wishes, should they lose the capacity to decide for themselves. However, by far the great majority of those who are competent to do so does not make such directives or give much thought to these issues. It is safe to say that, in Italy, the great majority of those who lapse into incapacity for any reason will not have issued prior directives. It is our contention that all adult and competent Italian citizens, while expressing their consent/dissent to the donation of organs as provided by law, would formulate prior directives specifically concerning EV. Prior to this time, the correct information should be provided to allow everyone to make an informed decision regarding this issue. Physicians and patients discuss their mutual values, those related to health and, in particular, those related to decisions about death and dying. The key question is whether the individual (potential donor) has the ability to understand, retain, believe, evaluate, weigh up, and use information that is relevant to a choice regarding organ donation and EV. Communication between patients and physicians requires language that conveys meaning and ensures understanding. Once the potential donor has expressed the wish to donate his/her organs and, if necessary, to accept EV, such a directive would be determining for physicians.

However, we are aware of the gravity of the perplexing problems surrounding the issue of EV. Respect for patient autonomy is one of a cluster of ethical principles that elevate the value of human life and is the basis for the decision-making process in medical practice; thus, the weighing and balancing of all basic principles of medical ethics have become an essential component of the reasoning process.

We are convinced that protecting the patient's autonomy and allowing that patient to attain the specific aim (organ donation) that he or she surely deems worthwhile place EV under the constraints of beneficence. In a modern view of medicine, we have the duty to interpret “benefit” in terms of the values or best interests of the patient, rather than in terms of strictly medical benefits. Traditionally, doctors have veered towards a “medicalized” perspective that has been heavily dependent on clinical judgment. The requirement for the determination of best interests encompasses a wider evaluation of the patient's concerns, of which the medical perspective is but one component. Other broader ethical, social, and moral considerations fall within the best interests of the person who freely chooses to donate organs [21]. Finally, strict rules are imposed by the Italian death statute, according to which physicians have the duty to accurately and reliably determine death. This ensures that the decision to withdraw extraordinary support is made without coercion from the transplant team waiting for the patient's organs and categorically excludes the possibility of any harm to the donor, thus leading to a wider acceptance of organ donation as a social practice. The certainty of death that the Italian law requires is a very strong guarantee for potential donors.

## 5. Conclusions

In Italy, there is no legal framework regulating the practice of EV, no real information is issued to the general public, and a public debate has yet to be initiated. Traditional moralists find it unacceptable that elective nontherapeutic ventilation and resuscitation are used to enable patients, for whom a decision to stop all therapy has been made, to evolve towards brain death and organ donation. They consider it to be disrespectful of the interests of the donor (patient), which is the medical mission (beneficence model); EV would violate both the principle of nonbeneficence to which Italian physicians are bound under the Code of Medical Ethics and the principle of patient autonomy [22]. However, an increasing affirmation of the principle of patient autonomy is pervading Italian law and policy, also through the growing ethical and legal value of patients' previously expressed wishes. Recently, some relevant decisions by the Italian Supreme Court dealt with the issue of advance directives, which still lack normative references in our legal system and are the subject of a lively and ongoing debate. Italian jurisprudence dealt with the problem of the relevance and validity of will previously expressed by a patient who, for pathological reasons, is no longer able to express such a will. What seems to be relevant is the predictability of the event, that is, that it is demonstrable that, even with the knowledge of what lies ahead, the patient stands by his/her decision and that all possible events have been foreseen.

In this ongoing cultural and legislative Italian scenario, we believe that, under some circumstances (i.e., the expressed wishes of the patient even in the form of advance directives), the use of EV could fall into the best interests of the patient and would thus not violate the principle of beneficence. The physician-patient relationship centers upon unique human experience; within this relationship the physician can help the patient to obtain specific aims, which that patient deems to be worthwhile. Surely the aims of this relationship must include morally and technically viable decisions made for, and with, the patient. The fusion of ethical and technical elements in clinical decisions has great relevance in the central question we are addressing. Technical and moral elements are not necessarily the same thing. The best interests of the patient may go beyond medical interests and may comprise the patient's values, goals, and beliefs beyond the narrow medical interest of health, cure, and prevention of illness and pain. The patient's values, wishes, and preferences underpin all his/her choices. Thus, when a competent citizen, according to Italian law, agrees to become an organ donor, is that citizen really oriented towards those actions that may facilitate the realization of his/her aim? We firmly believe that the crux of the matter lies precisely in this point, that is, the need to explore the real determination and will of the patient and his/her orientation towards the specific aim of organ donation.

We also note that it may not be appropriate to assume that all those that fail to opt out have no objection to becoming donors. This focuses attention on the urgent need to improve public awareness and understanding of organ donation in Italy. In this context, Italian physicians have yet to assume an important role as a source of information for citizens. A recent survey on knowledge and attitudes toward organ donation in Italy demonstrated that the Internet provides a considerable proportion of information sources (37.2%), compared to family doctors (5.6%) and school education (18.6%). Conversely, 68.5% of participants think that family doctors should provide information regarding donation and 81.9% think schools should also provide such an education [23]. Therefore, fair information and public awareness about organ donation are needed, and strong efforts must be aimed at involving Italian physicians in education about donation and EV.

This is the correct framework in which to evaluate the feasibility of EV in Italy and in which patients' previously expressed wishes become legally and morally pertinent. In this perspective, it appears to us essential to eliminate any ambiguity and emphasize that the patient's right to influence, even by means of advance directives, the treatment to which he or she might be subjected in the event of subsequent incompetence may also encompass the use of nontherapeutic ventilation for organ donation. The drawing up of advance directives must be developed and their scope extended to organ donation and elective resuscitation.
